# Live cell molecular analysis of primary prostate cancer organoids identifies persistent androgen receptor signaling

**DOI:** 10.1007/s12032-021-01582-y

**Published:** 2021-09-28

**Authors:** Erika Heninger, David Kosoff, Tamara S. Rodems, Nan Sethakorn, Anupama Singh, Harshitha Gungurthi, Kristin N. Carlson, Bing Yang, Cole Gilsdorf, Cheri A. Pasch, Dustin A. Deming, Leigh Ellis, David J. Beebe, David F. Jarrard, Joshua M. Lang

**Affiliations:** 1 University of Wisconsin Carbone Cancer Center, 1111 Highland Ave., Madison, USA; 2 Department of Medicine, University of Wisconsin, Madison, 1111 Highland Ave., Madison, WI 53705 USA; 3 Department of Urology, 1111 Highland Ave., Madison, WI 53705 USA; 4 Division of Medical Oncology, Department of Medicine, Cedars-Sinai Medical Center, Samuel Oschin Comprehensive Cancer Institute, 8700 Beverly Blvd, Los Angeles, CA 90048 USA; 5 Department of Pathology and Laboratory Medicine, 1111 Highland Ave., Madison, WI 53705 USA; 6 Wisconsin Institutes for Medical Research, Rm 7151, 1111 Highland Ave., Madison, WI 53705 USA

**Keywords:** Prostate cancer, PDCO, Primary culture, Microfluidics, Epigenetic biomarker

## Abstract

Prostate Cancer (PC) is a disease with remarkable tumor heterogeneity that often manifests in significant intra-patient variability with regards to clinical outcomes and treatment response. Commonly available PC cell lines do not accurately reflect the complexity of this disease and there is critical need for development of new models to recapitulate the intricate hierarchy of tumor pathogenesis. In current study, we established ex vivo primary patient-derived cancer organoid (PDCO) cultures from prostatectomy specimens of patients with locally advanced PC. We then performed a comprehensive multi-parameter characterization of the cellular composition utilizing a novel approach for live-cell staining and direct imaging in the integrated microfluidic Stacks device. Using orthogonal flow cytometry analysis, we demonstrate that primary PDCOs maintain distinct subsets of epithelial cells throughout culture and that these cells conserve expression of androgen receptor (AR)-related elements. Furthermore, to confirm the tumor-origin of the PDCOs we have analyzed the expression of PC-associated epigenetic biomarkers including promoter methylation of the *GSTP1*, *RASSF1* and *APC* and *RARb* genes by employing a novel microfluidic rare-event screening protocol. These results demonstrate that this ex vivo PDCO model recapitulates the complexity of the epithelial tumor microenvironment of multifocal PC using orthogonal analyses. Furthermore, we propose to leverage the Stacks microfluidic device as a high-throughput, translational platform to interrogate phenotypic and molecular endpoints with the capacity to incorporate a complex tumor microenvironment.

**Supplementary Information:**

The online version contains supplementary material available at 10.1007/s12032-021-01582-y.

## Introduction

Men with prostate cancer (PC) exhibit considerable diversity in clinical outcomes despite frequent similarities in cancer stage and treatments received. While the varied disease course is likely multifactorial, molecular profiling of PC biospecimens has revealed that the complexity and diversity of the proteo-genomic landscape amongst PC tumors may be an important driving factor in the different clinical trajectories [1]. Unfortunately, this molecular diversity is not reflected by the limited array of conventional monoclonal cell lines, which form the basis of most in vitro PC research. Such discord between the bench and the bedside may represent a major barrier to meaningful biologic discoveries as well as the development of novel translational therapies.

Three-dimensional organotypic models have emerged as an in vitro model system that can incorporate unique features of primary tumor biology, structural complexity, and intra- and interpatient tumor heterogeneity [2, 3]. In recent years, considerable improvements have been reported in the development of 3D organoid cultures in a variety of solid tumors, particularly in gastrointestinal carcinomas. The development of patient-derived cancer organoid (PDCO) models from primary PC tissue, however, has been halted by low processing efficiency, limited sample size, and lack of feasible technologies. Tissue biopsies yield few viable prostate epithelial progenitors and ex vivo senescence further limits lifespan of pre-metastatic prostate PDCOs [4]. Conventional analytic pre-processing like fixation, embedding and sectioning further reduces material and compromises biomarker patterns [5–7]. Furthermore, the scaffolding matrix makes it challenging to directly evaluate and manipulate these complex 3D structures. Although recent advances have been reported in propagation of hormone-naive PC PDCOs [4, 8], most data have been accumulated by molecular mapping or single-marker immunohistochemistry analysis, therefore, the sophisticated structure of primary PC PDCOs is yet to be explored in granularity to assess if they faithfully reflect native tissue heterogeneity.

In the current study, we aimed to perform a comprehensive orthogonal analysis of the cellular composition of PC PDCOs and compare content to their matched native tissue specimen. To achieve these ends, we developed a novel microscale approach for rapid evaluation of 3D PDCOs within an open microfluidic culture platform known as Stacks [9, 10], which enables in-chip buffer-exchanges and staining of live cells without material transfers and pre-processing. Due to the ultra-low volume of matrix in the open culture wells, this device also allows for direct high-throughput imaging. Additionally, we assessed PC-associated epigenetic biomarker patterns to confirm the presence of tumor cells in our primary specimens with a newly established microfluidic approach that enables screening of very-low-input analytes.

## Materials and methods

### Patients

Human PC tissues were obtained at the University of Wisconsin-Madison from patients undergoing radical prostatectomy who had received no prior treatments. The University of Wisconsin Institutional Review Board has approved utilization of all the tissue samples in this study, and written and informed consents have been obtained from all patients. Prostate tumor tissue was sampled by gross dissection from surgical prostatectomy specimens at the TSB Biobank at the University of Wisconsin Carbone Cancer Center.

### Prostate PDCO culture

Tissue samples from 4 mm diameter punch biopsy cores were sliced into ~ 1 mm diameter pieces and digested in 50 mg/mL collagenase (Sigma-Aldrich, MO #C9697) and 0.125 mg/ml dispase (Invitrogen) for up to an hour in a 37 °C water bath under close observations, including vortexing and vigorous manual shaking every 5–10 min. Digestion was stopped when the bulk of the tissue visually disintegrated and well-defined spheres occurred in the homogenate. The digested tissue was washed three times in PrEGM media (Lonza) and the pellet was resuspended with fine pipette tips until the homogenate was broken up. Spheres generated from the tissue digest were resuspended in PrEGM media supplemented with 0.1 mg EGF and 10 mM Y-27632. Samples were seeded with a density of ~ 5–10 spheres per well onto a 24-well plate in 50% BD Matrigel™ GFR (BD Biosciences, CA) in hanging droplets. Once the hydrogel solidified, culture plates were inverted and cultures were fed with PrEGM media supplemented with 10 uM Y-27632 (Sigma-Aldrich, MO) every three days. Cultures were visually monitored for PDCO growth, viability and structural integrity. Cultures were passaged if exceeding optimal density or contained excessive amount of tissue debris.

### Stacks microfluidic device and drug cultures

The Stacks device [9, 11] (injection molded by Proto Labs Inc., MN) was cleaned and sterilized by brief ultrasonication in a water bath followed by soaking in 70% ethanol, air-drying and exposing to UV-light for 15 min each side in a biosafety cabinet. PDCOs were resuspended in 4.5 ul 50% BD Matrigel™ GFR and transferred to Stacks wells at a density of ~ 2–5 per well. The device was then placed in a sterile humidified chamber at 37 °C for 15 min until the matrix solidified. A droplet of 10 ul of PrEGM media was then pipetted on top of each culture well and cultures were incubated at 37 °C with 5% CO_2_ until further processing. For drug treatment studies, 10 nM Docetaxel (Selleckchem, TX) or 100 uM Bicalutamide (Chemietek, Indianapolis, IN) or DMSO (Fischer Scientific, MA) was added to media for three days. An internal control for dead cell staining was generated by treating PDCO samples with 1% PFA for 15 min prior to washing with media three times and staining.

### Fluorescent microscopy

Immune fluorescent labeling was performed by leveraging microfluidic fluid dynamics in the Stacks device. Culture wells were incubated with a 10 ul droplet of PrEGM media containing the relevant combination of monoclonal antibodies (Online Resource Table S1), Hoechst33342 and IMAGE-IT™ DEAD Green™ (ThermoFisher Scientific, MA for the two later reagents) viability marker at 37 °C. The samples were then washed with three volumes of PrEGM media following fixation with 1% PFA for 30 min and rinsing 3 times. Intracellular staining for androgen receptor (AR) and prostate specific antigen (PSA) protein expression was done following permeabilization with 0.1% Tween 20 in PBS for 15 min and three washes. Stacks wells were then mounted with a glass coverslip with Slow Fade™ Gold Antifade mounting media (ThermoFisher Scientific, MA) and imaged on a Nikon W1 Spinning-disc confocal microscope followed by image analysis with the NIS-Elements microscopy imaging software.

### Flow cytometry analysis

Native tissue was cut into ~ 1 mm diameter pieces and digested in 50 mg/ml collagenase and 1000 U/ml DNase I for up to 2 h in a 37 °C water bath, mixed vigorously every 10–15 min and monitored closely until the bulk of tissue disintegrated into single cell suspension. The samples were then resuspended with repeated pipetting using a 1000 ul tip followed by processing through a 70 mm cell strainer. PDCOs were spun out of the matrix followed by digestion with Trypsin–EDTA (HyClone, Thermo Fisher Scientific) and resuspension in BD Pharmingen™ Stain Buffer (BD Biosciences, CA).

Cells were stained with Ghost Dye™ Violet 510 fixable live/dead stain (Tonbo Biosciences, Sand Diego, CA) and fluorescently labeled antibodies listed in Online Resource Tables S2 and S3. For intracellular staining, fixation and permeabilization was performed following the manufacturer’s protocol (eBioscience, Thermo Fisher Scientific, MA). Samples were acquired following a pre-acquisition instrument standardization with SPHERO™ Ultra Rainbow Fluorescent Particles (Spherotech, IL) on a BD LSR II instrument at the UWCCC Flow Cytometry Laboratory and data were analyzed by FlowJo v9.6 (BD Biosciences, CA). Gating controls included Internal Negative Controls (INC) and Fluorescent Minus One (FMO) controls.

### Epigenetic screening of prostate tissue specimen

#### Methylated DNA enrichment

Methylated DNA enrichment was performed using the SEEMLIS [**S**emi-Automated **E**SP (Exclusion-Based-Sample Preparation) **E**nrichment of **M**ethylated DNA from **L**ow **I**nput **S**amples] method as follows. PDCO or tumor biopsy DNA was extracted using the AllPrep DNA/RNA Micro kit (Qiagen) according to manufacturer’s instructions. DNA was quantified on a Qubit and 1-10 ng was digested using 1 ul of each restriction enzyme (AluI and HpyCH4V; NEB) in 20 ul reactions containing 1 × Cutsmart Buffer (NEB) for 15 min at 37 °C followed by enzyme inactivation for 20 min at 80 °C. Samples of equal amounts of LNCaP and white blood cell (WBC) DNA were included as positive and negative controls, respectively. Twenty-five ul of TALON magnetic beads (Takara) were washed 3 × with 100 ul 1 × Binding Buffer (BB) (4% glycerol, 1 mM MgCl_2_, 0.5 mM EDTA, 120 nM NaCl, 2 mM Tris–HCl pH 7.4, 0.2% Tween-20, and 0.5 mM DTT). Washed beads were resuspended in 100 ul MBD2-MBD Coupling Buffer [1 × BB, 1 × Halt protease inhibitor cocktail (Thermo Fisher), 500 ng Unmethylated Lambda DNA (Promega), 5ul his-tagged MBD2-MBD (EpiXplore Kit, Takara)] and placed on shaker at RT for one hour to bind MBD2-MBD to the TALON beads. MBD2-MBD bound beads were washed 3 × with 100ul 1 × BB and resuspended in 88µL 1 × BB with 1 × Halt protease inhibitor cocktail and added to 20 µl restriction enzyme digested DNA in 200 µL PCR tubes. This reaction was placed on a shaker at RT for three hours to bind methylated DNA to MBD2-MBD-coated TALON beads. PCR tubes were placed onto Gilson PIPETMAX® liquid handling robot for ESP enabled washing and elution steps. The whole elution volume of approximately 12.5 ul including beads was manually pipetted into new 200 ul PCR tubes containing pre-amplification reagents. Pre-amplification was performed using custom TaqMan probes and TaqMan PreAmp Master Mix (Thermo Fisher) according to manufacturer’s specifications. Volumes indicated are per reaction.

#### Quantitative real-time PCR and pre-amplification

Quantitative RT-PCR was performed using TaqMan probes (ThermoFisher) and iTaq Universal Probes Supermix (Bio-Rad). Custom probes were used for methylation analysis and primer and probe sequences are listed in Online Resource Table S4. Cycling conditions: five minutes at 95 °C for initial denaturation and enzyme activation followed by 45 amplification cycles of 5 s at 95 °C and 30 s at 60 °C. Cycling conditions for pre-amplification: 10 min at 95 °C for enzyme activation followed by 14 cycles of 95 °C for 15 s and 60 °C for four minutes. Pre-amplified samples were diluted 1:5 with TE. LINE1 primers were not included in pre-amplification mix.

#### Data analysis and calculation methods

Enriched methylated DNA Ct values for PDCO and biopsy samples and controls were normalized to positive control Ct values. LNCaP DNA was used as a positive control for *GSTP1*, *RASSF1*, *APC*, and *RARB* promoter methylation. A Methylation Index from 0.0 to 1.0 was calculated for each sample using LNCaP methylation as 1.0 on the Methylation Index scale. Raw Ct values were converted to relative values using the delta Ct method and then divided by the relative methylation in LNCaP cells as follows:

$${\text{Methylation}}\;{\text{Index}} = \frac{{2^{{ - {\text{Ct}}_{{\text{Spher/Biop}}} }} }}{{2^{{ - {\text{Ct}}_{{{\text{LNCaP}}}} }} }}$$

Relative *LINE1* methylation was determined relative to the cycle limit (45 cycles) using a modified form of the delta-delta Ct method as follows:

$${\text{Relative}}\;{\text{Methylation }} = 2^{{ - \left( {{\text{Ct}} - 45} \right)}}$$

### Statistical analysis

Statistical analysis for the PDCO drug treatment experiments was performed by ordinary one-way ANOVA with the Holm-Sidak’s multiple comparison test.

## Results

**Fig. 1:**
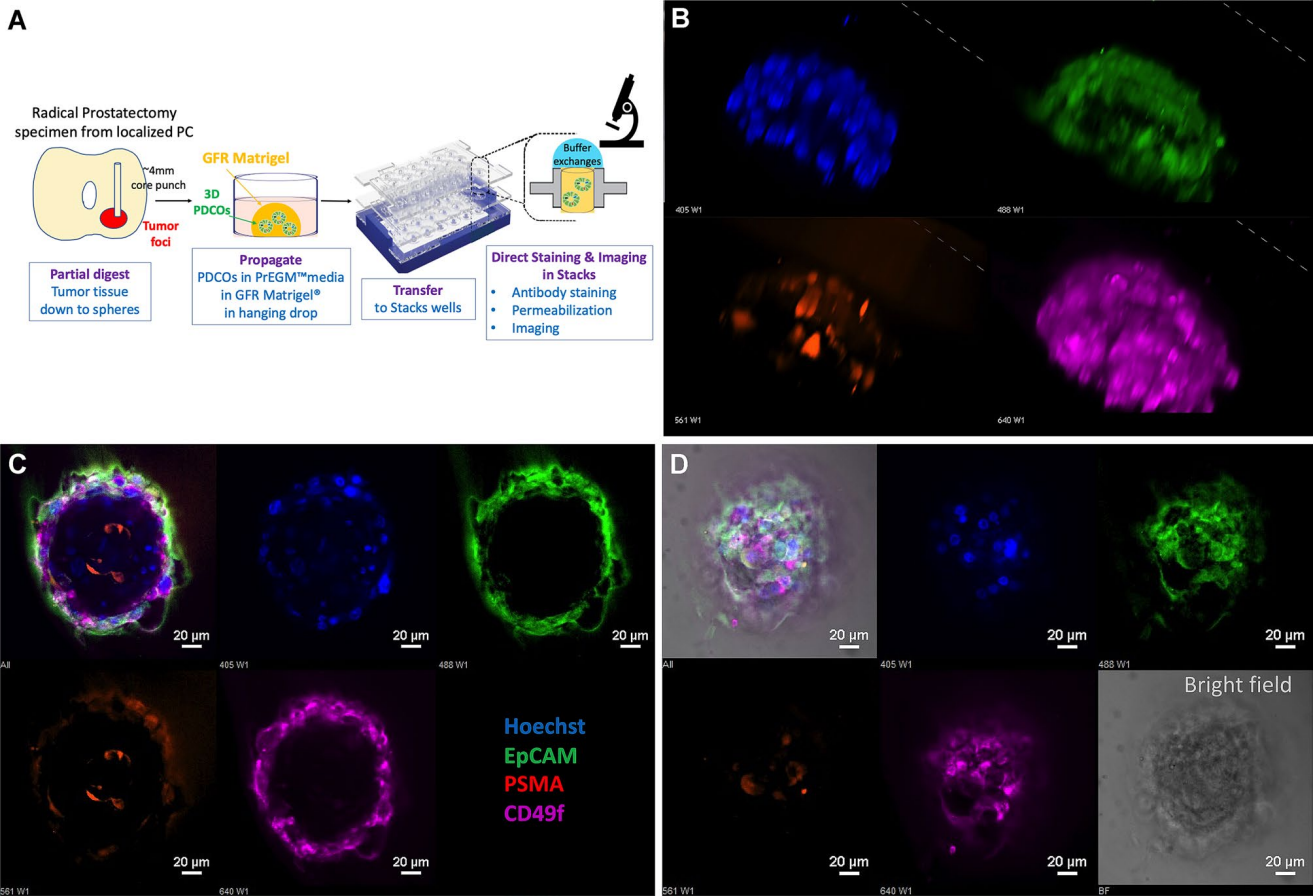
Live staining and direct confocal imaging of primary prostate carcinoma-derived PDCOs in the Stacks microfluidic device. **A** Schematic outline of PDCO culture processes. PDCO structures were raised ex vivo from radical prostatectomy punch biopsy. At around day 10–14, the PDCOs were transferred to the Stacks culture device and subjected to live staining followed by direct confocal imaging in-chip. PDCOs were stained with Hoechst (blue), EpCAM (green), PSMA (red) and CD49f (purple). **B** Structure of a prostate PDCO as condensed 3-dimensional images in single channels. **C** Mid-section image of the same structure as merged (upper left) and individual channels. **D** Top section of the same PDCO including individual channels, a brightfield image (bottom right) and merged (top left)

Between January and December of 2019, 48 donor specimens were collected from patients undergoing radical prostatectomy for high-risk, organ-confined prostate carcinoma. Following gross examination, a ~ 4 mm diameter biopsy core punch was collected. The tissue samples were subjected to partial digestion and the preparations were cultured as described in the Methods Section (Fig. 1A). Out of the 48 biopsy specimens, 23 produced three-dimensional PDCOs with an overall success rate of 47.9% (Table 1). Cultures were harvested at their peak which was determined by observations of the culture dynamics and PDCO developmental status. We assessed viability by observing morphological features, growth and structural integrity. PDCOs were also screened for re-differentiation into 2D growth. Peak culture time was determined to be the stage of growth before signs of cell death, structural disintegration and 2D re-differentiation were observed in the culture. The median peak and harvest of cultures was at day 16, with a range of 10–24 days.

**Table 1:** Prostate PDCO specimen enrolled in PDCO Culture Studies between January and December of 2019

2019 Jan to Dec	
Median Spheroid Culture Peak Day	16 days (10–24)
Total samples plated in 2019	48
Successful 3D growth	23
Low 3D yield < 12 spheroids	7
Overall success rate	47.9%

### Microfluidic stacks platform allows for live staining and direct imaging of primary prostate carcinoma-derived PDCOs

To establish a logistically feasible, comprehensive approach to analysis of the 3D PDCO structures from small sample size without pre-processing and material transfer, we developed a simplified workflow for immune fluorescent labeling of live PDCOs and in-chip imaging utilizing the integrated analytic capabilities of the microfluidic Stack device [9, 11]. Microscale fluid dynamics in the Stacks wells allow for rapid buffer exchanges between the hydrogel and a 10 ul volume buffer droplet on top enabling in-chip immune fluorescent labeling without material transfers. At 10–14 days in culture, PDCOs were harvested from the hydrogel matrix and transferred into Stacks culture wells in 50% Matrigel™. Antibody staining was performed via buffer exchanges in-chip. After staining, the device was directly mounted and cells were visualized within the Stacks culture wells using confocal microscopy (Fig. 1A).

Through confocal imaging we were able to clearly visualize the PDCO structures within the Stacks culture wells (Fig. 1B). We observed that most PDCO structures were organized in a dome-shaped structure with an opening on the opposite side (Online Resource Figure S2). Multi-channel interrogation has demonstrated that the PDCO structures primarily consist of cells that co-expressed the EpCAM (Trop1, in green) pan-epithelial antigen and CD49f (alpha6-integrin, in violet) (Fig. 1C, D). Both surface proteins are conventionally expressed in prostate epithelial cells and are associated with carcinoma origin [12, 13]. Prostate specific membrane antigen (PSMA) expression was also detectable in these primary structures, further confirming the prostate epithelial origin of the cellular content.

### The cellular composition of primary prostate tissue-derived PDCO structures retains epithelial dominance

**Fig. 2:**
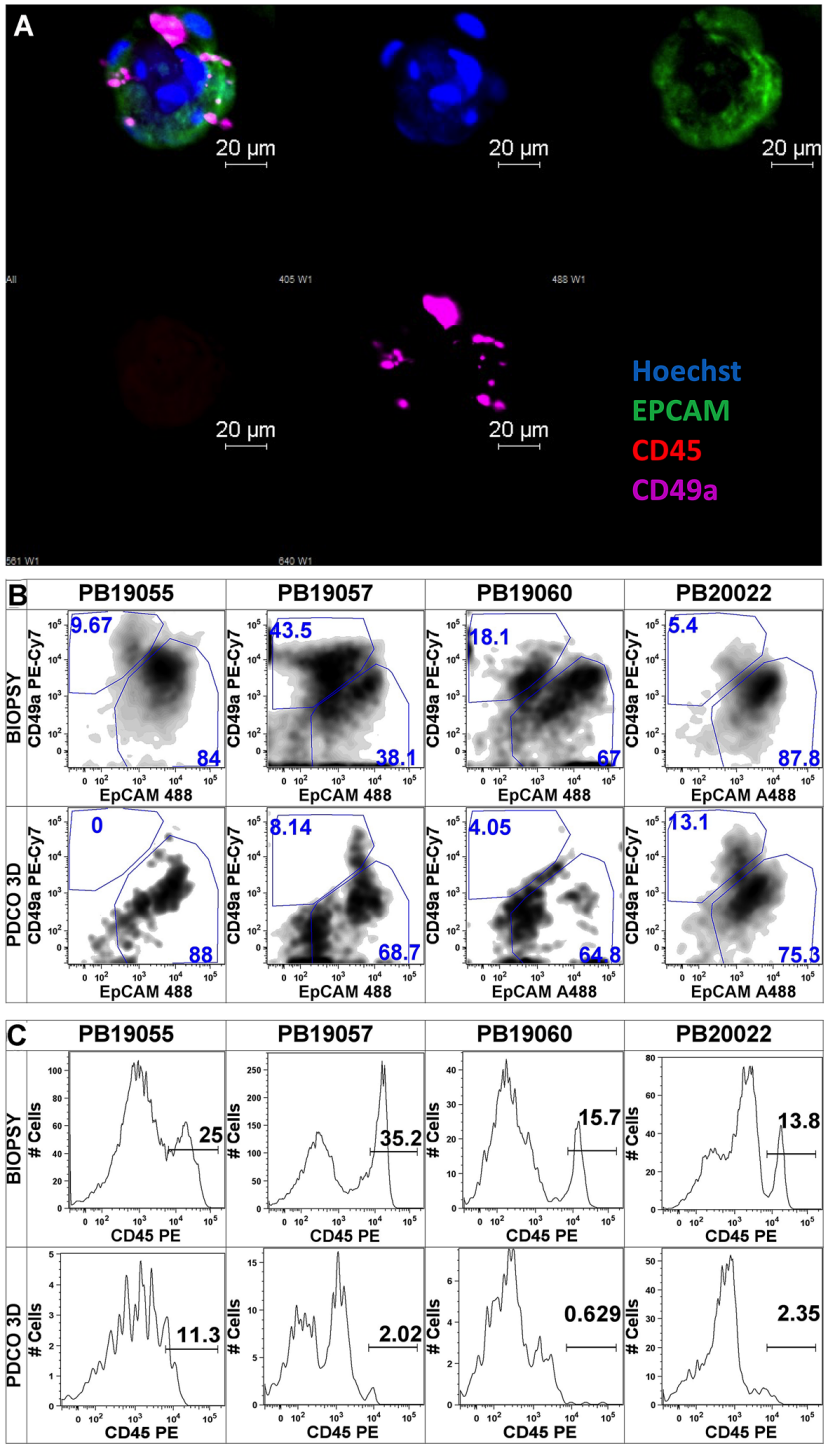
Ex vivo propagated primary prostate carcinoma PDCOs retain prostate epithelial dominance. **A** Patient-derived primary PDCO structures were subjected to live staining with Hoechst (blue), EpCAM-Alexa488 (green), CD49a-Alexa647 (purple) and CD45-PE (red) followed by direct confocal imaging in Stacks wells. The upper left block shows a merged image of a mid-section of the structure. The other images represent individual channels. **B** Flow cytometry analysis of matched samples of four individual patients that were harvested at day 0 (biopsy) and at day 10–14 of the ex vivo PDCO culture (PDCO 3D). Density plots represent frequency of EpCAM^+^ and CD49a^+^ cells after exclusion of debris, aggregates, dead cells and CD45^+^ cells. **C** Flow histograms representing the CD45^+^ content of total cells in prostate PDCOs

Previous reports of PDCO cultures initiated from primary PC have reported technical challenges including a potential overgrowth of tumor-associated spindle cells. We, therefore, interrogated our primary specimen for the presence of three major prostate tissue microenvironment (TME) components: (1) epithelial, (2) stromal and (3) immune cells. To achieve these ends, we harvested PDCOs at day 10–14 following ex vivo culture and passaged to Stacks device for immune fluorescent labeling and imaging. We stained the live samples with anti-EpCAM (shown in green), anti-CD49a (stromal marker, shown in violet) and anti-CD45 antibodies (leukocyte marker, shown in red) (Fig. 2A). Confocal imaging demonstrated that the PDCOs were dominated by EPCAM^+^ epithelial cells and contained some CD49a^+^ stromal cells (bottom row, middle block). Most PDCO structures contained only sporadic CD45^+^ leukocytes, which was expected as the culture conditions did not include stimuli supporting immune cell survival such as leukocyte growth factors or antigens. Next, we investigated, whether the cellular composition of the PDCOs changed over time during culture as compared to the starter native tissue preparations. For this analysis, we obtained two punch core donations per donor from four individual donor patients. We cut the cores into ~ 1 mm cubes, pooled the tissue pieces and divided the subsequent sample in two portions for separate enzymatic processing of PDCO cultures and for flow cytometry. At day 10–14 of culture, PDCOs were harvested and dissociated with a brief enzymatic digestion and single cell suspensions were analyzed by flow cytometry. To synchronize fluorescent read-outs between time-points, instrument settings were adjusted using Ultra-Comp Mid-Range Rainbow beads prior to acquisition. Gates were established using Internal Negative Control and Fluorescent Minus One controls shown (Online Resource, Figure S1). Density plots representing the single live cells (Fig. 2B) demonstrate that the CD49a^+^ stromal content was present in the PC tumor tissue isolates; however, the frequency of these cells decreased over time under ex vivo culture conditions and the cellular composition of 3D PC PDCOs was dominated by EpCAM^+^ epithelial cells. Leukocytes were detected as a minor component in the biopsy tissue (Fig. 2C) as well. As expected, following ex vivo PDCO culture, the number of CD45^+^ events further reduced in accordance with our confocal imaging observations.

### Primary ex vivo prostate PDCO cultures contain a variety of epithelial subsets and reflect patient heterogeneity of original tissue

The intratumor heterogeneity of prostate epithelial cells in multi-focal PC has been previously established and variable expression levels of various epithelial biomarkers including CD49f and TROP2 were found to define distinct epithelial subsets within tumor foci [12, 14–17]. We aimed to assess whether the cellular heterogeneity observed in the epithelial compartment of native prostate tissue is maintained following ex vivo propagation. We compared the composition of the EpCAM^+^ subset in our primary prostate isolates before and after ex vivo culture at day 0 and then at day 10–14 in culture, respectively.

**Fig. 3:**
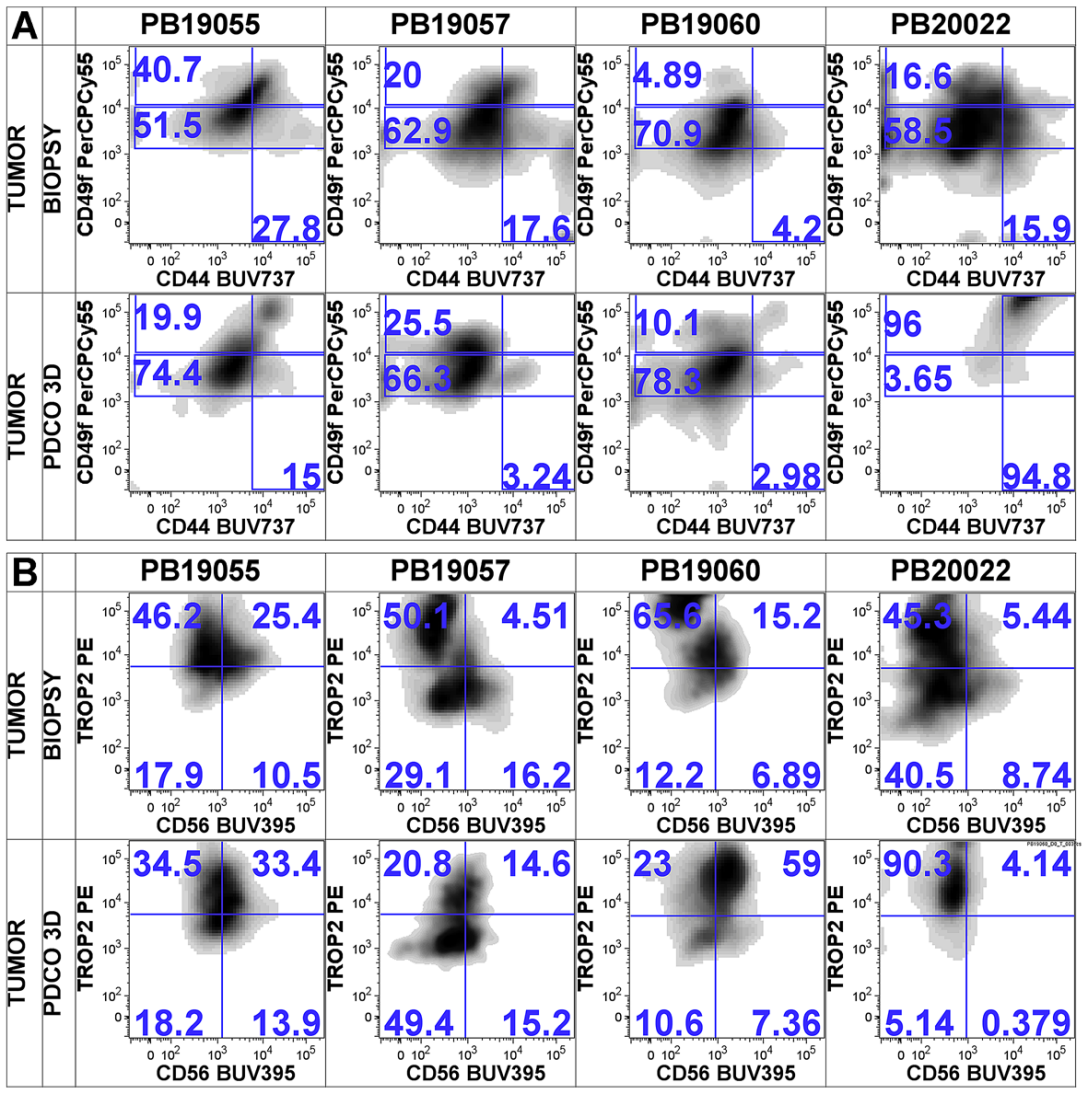
The cellular composition of primary prostate PDCOs reflects a variety of epithelial subsets and inter-patient heterogeneity. **A** Density plots represent flow cytometry analysis of matched samples of four individual patients. Numbers represent subset frequency within the EpCAM^+^ cells. CD49f^bright^ (upper left), CD49f^intermediate^ (lower left) and CD44^+^ (lower right). **B** Density plots represent TROP2 and CD56 expression within of the total EpCAM^+^ cells

We measured the surface protein expression of CD49f and TROP2 on the EpCAM^+^/CD45^−^/CD14^−^ epithelial subset (Fig. 3A). CD49f has been previously proposed as a robust biomarker of prostate tumor cells with superior tubule-forming capacity under ex vivo conditions[18, 19]. We observed a heterogenous distribution of CD49f^high^ and CD49f^int^ subpopulations in native tissue specimens, and this distribution remained similar after ex vivo propagation in most cultures. This suggests that both CD49f^high^ and CD49f^int^ subsets may contribute to 3D sphere formation in our protocol. Interestingly, in PB20022 we observed a shift in CD49f expression with a dominant CD49f^high^ population and loss of heterogeneity in CD49f expression. We found 16.6% CD49f^high^ and 58.5% CD49f^int^ in the biopsy and 96% CD49f^high^ and 3.65% CD49f^int^ in the 3D PDCO culture, respectively. This suggests that a pre-dominant CD49f^high^ subset was likely present in this particular specimen that outgrew other subpopulations.

CD44 has been previously associated with more aggressive disease pathogenesis and poor clinical outcomes in PC [20]. It has been shown to enrich in stem-cell-like prostate cells. We analyzed CD44 expression levels in our primary prostate specimens before and after PDCO culture (Fig. 3A). We have identified a minor subset of EpCAM^+^ cells that expressed CD44 and this subpopulation remained present in similar frequencies following ex vivo propagation in most PDCO cultures. We found no evidence of a general change or bias in CD44 expressing epithelial cells in the PDCO cultures. However, in PB20022, we found a robust shift of CD44 overexpression in the subset that dominated the PDCO growth (15.9% at day 0 vs 94.8% at day 10 in culture). This same population also overexpressed CD49f suggesting a predominant stem-like subset that propagated in this particular ex vivo culture.

Human trophoblast cell surface antigen (TROP2) has differential expression in normal epithelial tissue and is overexpressed in prostate carcinoma [21, 22]. In the native tissue specimen, we observed variable TROP2 expression patterns within the EpCAM^+^ subsets. Furthermore, we found that the distribution of TROP2^bright^ and TROP2^low^ cells in most PDCO cultures reflected the original TROP2 expression patterns detected in the biopsy tissue (Fig. 3B). In PB20022, however, we detected a shift to TROP2 overexpression that also coincided with the increase in CD44 and CD49f expression.

Neuroendocrine cells are present in the prostate gland as a minority and can be identified as epithelial cells that express the CD56 (NCAM) biomarker. We were interested in assessing the neuroendocrine component and we analyzed if they were maintained under ex vivo conditions. We have detected CD56^+^ cells in the EpCAM^+^ subset that represented the minority of epithelial cells in most biopsies and remained detectable after ex vivo propagation (Fig. 3B).

### Prostate PDCO cultures maintain AR pathway activity

**Fig. 4:**
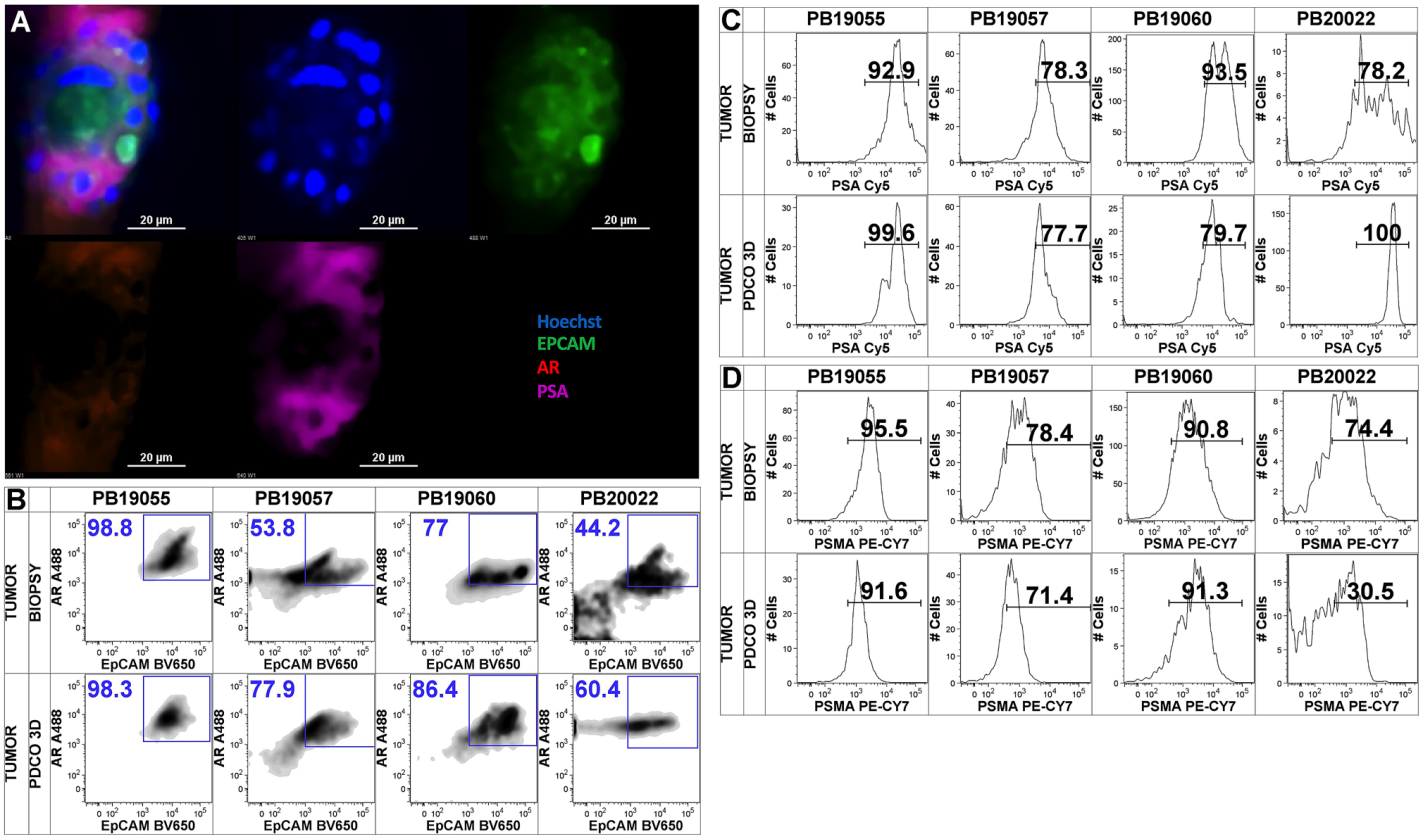
Ex vivo propagated primary prostate carcinoma PDCOs retain expression of AR-related proteins. **A** Patient-derived primary PDCO structures were subjected to live staining with Hoechst (blue) and EpCAM-Alexa488 (green). After fixation and permeabilization, the PDCOs stained with anti-AR, secondary anti-IgG-PE (purple) and PSA-Cy5 (red) followed by direct confocal imaging in Stacks wells. The upper left block shows a merged image of a mid-section of a representative PDCO structure and other images represent individual staining channels. **B** Flow cytometry analysis of matched samples of four individual patients generated at day 0 (biopsy) and at day 10–14 of the ex vivo culture (PDCO 3D). Density plots represent the frequency of EpCAM^+^ and AR^+^ cells of the total live, singular, cellular component after exclusion of CD45^+^ and CD14^+^ immune cells debris, aggregates and dead cells. **C**, **D** Histograms represent the expression spectrum and frequency of PSA^+^ and PSMA^+^ events within the EpCAM^+^/CD45^−^/CD14^−^ subset

Previous studies of prostate carcinoma-derived primary spheroid cultures have reported a potential alteration of the molecular biomarker pattern that may include a decline in both AR gene activity and the expression of AR-driven prostate biomarkers [4, 8]. AR activity is a key element in driving early PC pathogenesis. To assess activity of the AR-signaling pathway under ex vivo culture conditions, we measured AR, PSA and PSMA protein expression in PDCO specimen. First, we transferred live PDCOs to Stacks culture wells to evaluate AR-pathway activity by direct in-chip intracellular immune fluorescent labeling and confocal image analysis. We analyzed expression of two downstream signature proteins including AR and PSA. We detected expression of both AR protein (Fig. 4A, red staining) and PSA (Fig. 4A, violet staining) in the 3D PDCO structures indicating functional AR-pathway activity maintained under ex vivo conditions. To determine if there is any change in AR-pathway activity during propagation in our model, we measured AR, PSA and PSMA protein expression from single cell suspensions generated at the time of harvest and from matched PDCO cultures. The density plots in Fig. 4B show AR-expression of the EpCAM^+^/CD45^−^/CD14^−^/single/live cellular content of the tissue homogenates (biopsy, top row) and their matched PDCO-derived samples (PDCO 3D, bottom row). The data demonstrates that AR-expressing epithelial cells dominated the specimen both before and after culture. Next, we measured the intracellular PSA expression of the EpCAM^+^ cells in our matched samples and found that the frequency of PSA^+^/EpCAM^+^ double-positive cells was comparable before and after culture (Fig. 4C). PSMA, another AR-driven prostate biomarker was also detectable in PDCOs and the frequency of PSMA^+^/EpCAM^+^ cells remained comparable to levels detected prior to ex vivo propagation (Fig. 4D).

### Rapid microscale assessment to establish the presence of prostate carcinoma in low-volume primary tissue-derived samples

**Fig. 5:**
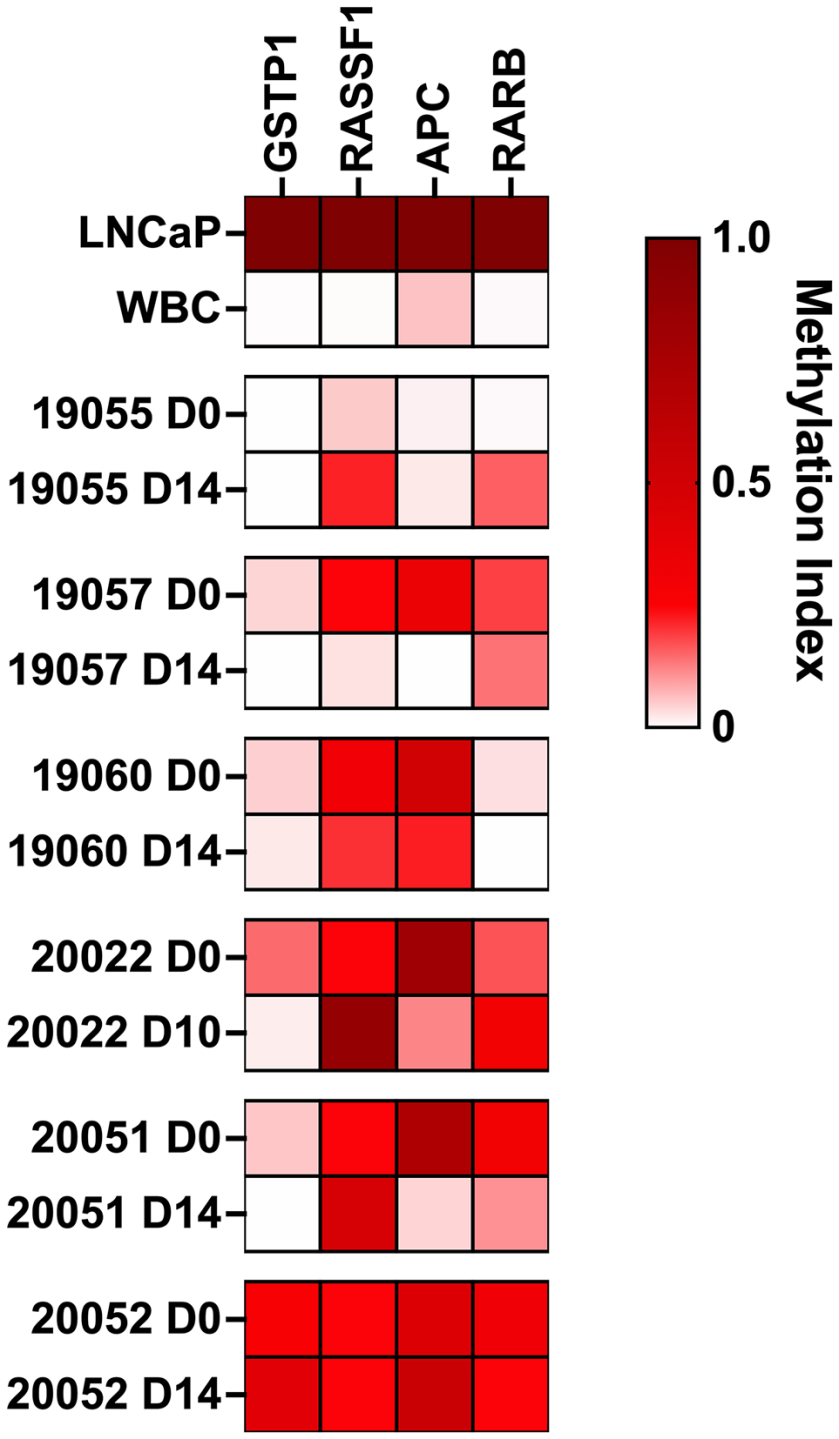
Microscale screening of prostate carcinoma related epigenetic biomarkers. A micro-sample of ~ 100–1500 cells was harvested from single cell homogenates from biopsy samples at day 0 and their matched PDCO cultures at day 10–14 from 6 individual biopsy core punches of radical prostatectomy specimen. DNA was enzymatically fragmented and methylated DNA was enriched with MBD-coated magnetic beads in an automated VERSA microfluidic system. The samples were subjected to qPCR to detect *GSTP1, RASSF1, APC*, and *RARB* genes. LNCaP cells were analyzed as a positive control, peripheral donor CD45^+^ white blood cells (WBC) were used to represent hypomethylated samples. The data represents matched biopsy and PDCO samples from individual patients

Successful establishment of PDCO cultures from organ-confined primary PC requires an optimal amount of tumor content and overgrowth of normal epithelia and tumor-associated spindle cells has been previously reported in primary PC-derived ex vivo cultures diluting tumor biomarkers [4, 23, 24]. Therefore, it is important to assess cancer cell content in these cultures. Traditional sampling of PC tumor foci is based on gross examination that has reportedly low accuracy [25] and conventional screening methods like the Prostate Triple Antibody Stain [26] further reduces precious material. To confirm cancer-cell content in our low-volume primary tissue specimen, we have developed a multiplex epigenetic biomarker assay to detect promoter methylation of PC-associated genes including *GSTP1*, *RASSF1*, *RARb* and *APC* [27–31]. We collected matched samples of ~ 100–1500 cells from single cell suspensions generated from native biopsies and subsequent prostate PDCO specimens, isolated total DNA and performed and MBD-based enrichment of methylated DNA using the SEEMLIS method on the semi-automated VERSA microfluidic platform [32]. We then performed quantitative PCR analysis to interrogate the promoter-methylation of selected PC-associated genes (Fig. 5). We included the LNCaP cell line as a positive control and CD45^+^ peripheral blood-derived white blood cells (WBCs) from a patient with PC as a negative control. Methylation index was calculated for each gene by normalizing sample signal to LNCaP signal as described in the Methods section. *LINE1* methylation was used as a control for successful enrichment of methylation (Online Resource, Figure S3a). Relative *LINE1* methylation tended to decrease in PDCO cultures compared to matched biopsy samples, which is likely due to the reduced level of *LINE1* methylation present in PC cells compared to healthy cells and may indicate the presence of a higher percentage of PC cells in our PDCO cultures relative to biopsy samples. A heat map was generated using Methylation Index values (Fig. 5). Methylation index values are also shown in bar graph format in Online Resource, Figure S3b.

Promoter methylation was detected above WBC level in at least two genes in all biopsy and PDCO samples, indicating the presence of prostate tumor cells in the biopsies and PDCO cultures. The level of *GSTP1* methylation was low compared to LNCaP cells, however, all biopsy (D0) samples except 19055 had *GSTP1* methylation at levels higher than WBCs. Interestingly, PDCO samples 20051 and 19057 lost *GSTP1* methylation but retained at least two other PC methylation markers. All samples had *RASSF1* methylation levels greater than WBC. *APC* methylation tended to decrease in PDCO cultures compared to matched biopsies. Three out of six pairs (19060, 20022 and 20052) had *APC* methylation higher than WBC in both the biopsy and PDCO samples. *APC* methylation dropped to below WBC levels in the PDCO cultures of 19057 and 20051 but both were above WBC levels in their matched biopsy samples. Only one out of six specimen (19055) did not show *APC* methylation above WBC levels in either the biopsy or PDCO culture. All samples had *RARB* methylation above WBC levels except the 19055 biopsy sample and 19060 PDCO sample. Overall, we detected promoter methylation of at least one PC-associated biomarker in all primary prostate specimens above WBC levels suggesting the presence of tumor content in the samples.

### Drug treatment of primary prostate PDCO cultures

**Fig. 6:**
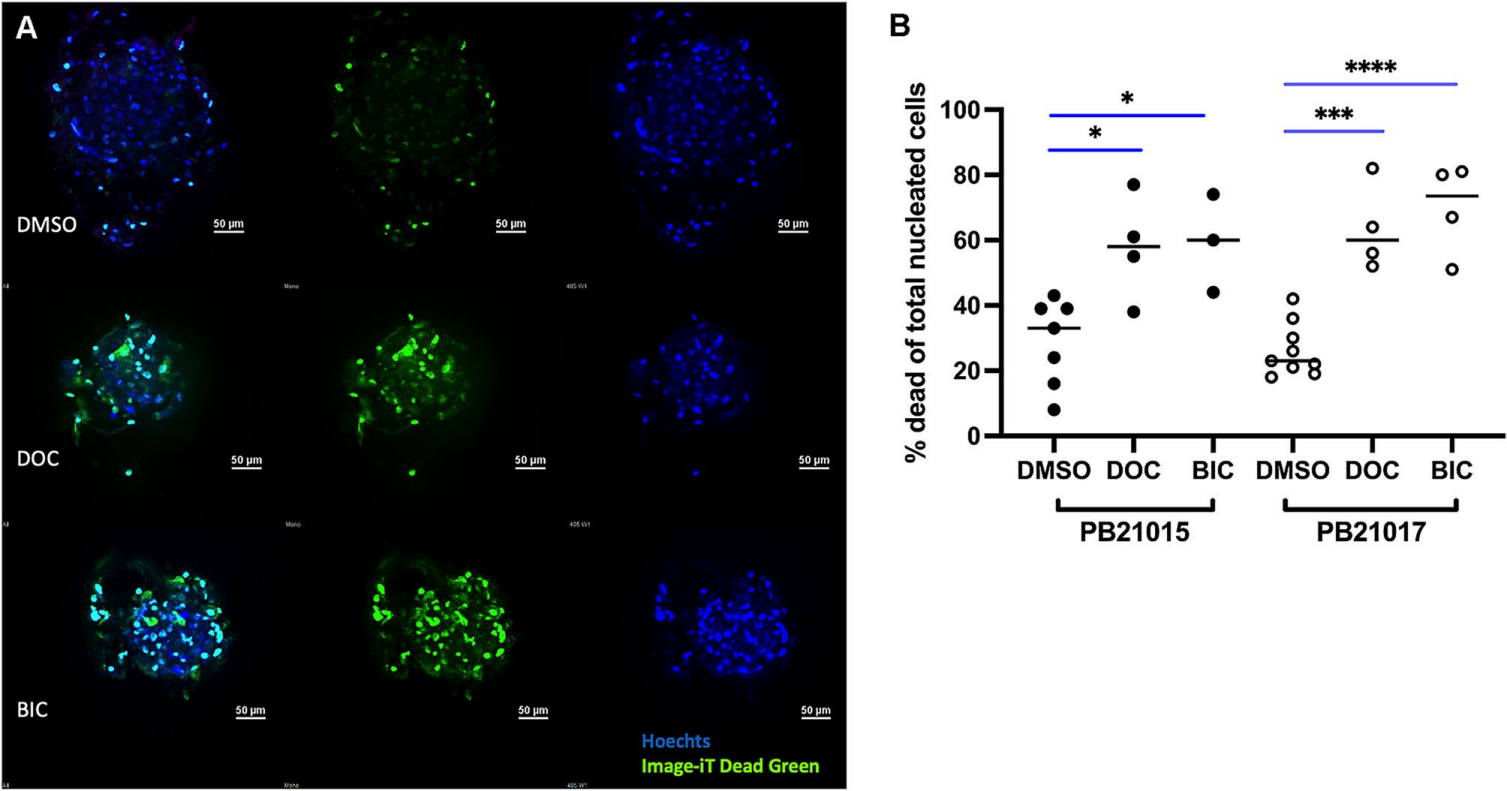
**A** Fluorescent images of prostate PDCOs after three days treatment with DMSO, Docetaxel (DOC) or Bicalutamide (BIC) in representative patient cultures. Nuclear staining (Hoechst, blue), Image-IT™ DEAD Green™ stain (green). **B** The frequency of dead cells is expressed as number of cells that accumulated live/dead stain in nuclei projected to the total nucleated counts in each individual PDCO structure. Data points represent individual PDCOs generated from two independent patient specimens. *p < 0.05, ***p < 0.001, ****p < 0.0001

To assess if Docetaxel or Bicalutamide influence primary prostate PDCO cell viability, we transferred patient-derived PDCOs to Stacks culture wells and treated them with 10 nM Docetaxel (DOC) or 100 mM Bicalutamide (BIC) or DMSO. After three days, we assessed cell death within the PDCO structures by direct fluorescent staining and confocal image analysis (Fig. 6A). The number of dead cells in each individual sphere was manually spot counted. Percent of dead cells was projected to the total number of nuclei within each individual whole PDCO (Fig. 6B). Bicalutamide treatment resulted in a significant increase in dead cell frequency in both model patients. DMSO vs BIC, in PB21015, 33% vs 60%, *p* = 0.0202 and in PB21017, 23% vs 73.5%, *p* < 0.0001, respectively. Docetaxel treatment increased cell death in PB21015 from 33 to 58%, *p* < 0.016 and in PB21017 from 23 to 60% *p* = 0.0001, respectively.

## Discussion

Recent advances in organotypic culture techniques have enabled ex vivo propagation of tumor-derived PDCOs that incorporate primary cell biology, 3D structural complexity, and tumor heterogeneity into in vitro model systems [2, 4, 16, 23, 33–37]. The conventional approach to organoid analysis includes traditional tissue processing like FFPE and sectioning [4, 23, 24]. However, these techniques may hamper analysis due to sample loss, molecular cross-linking that results in irreversible changes of organic matrix and diminished epitope availability compromising biomarker detection [5–7]. In the current study, we developed a microscale approach that permits direct interrogation of native biomarker expression patterns on live PDCOs. We then utilized this approach to perform a comprehensive assessment of primary prostate PDCOs from patients with localized PC.

We found that, following ex vivo culture, the PDCOs were dominated by epithelial cell populations. While the epithelial cells were also the primary cell type identified in the biopsy specimens, the proportion of other populations, such as stromal and immune cells, decreased during culture resulting in a greater predominance in epithelial cells over time. Surface marker analysis of these epithelial cells at biopsy demonstrated distinct cellular subsets, which is consistent with the well-documented heterogeneity identified in prostate tumors. We were able to demonstrate that this epithelial heterogeneity was generally maintained throughout the 10–14 day culture, further confirming the role for PDCO culture in incorporating and investigating tumor heterogeneity in vitro. Previous studies have demonstrated that certain epithelial subsets, including CD44^+^, CD49f^+^, TROP2^+^ cells, have the potential to predominantly drive sphere-forming activity [14, 19]. In our model, we did not observe a particular subset with greater proliferation or survival in the 3D PDCO culture, but rather a preservation of the intra-tumoral heterogeneity of epithelial subpopulations observed in the original tissue specimens in most matched cultures. However, one potential limitation to our approach is that we did not directly assess for basal cell outgrowth following ex vivo culture of PDCOs. Dominant basal outgrowth is a pervasive challenge in ex vivo organoid culture and the occurrence of this process can dramatically alter the composition of organoids. While our data, which did not demonstrate dominant expansion of any specific epithelial subset, suggests that basal outgrowth did not occur in our PDCOs, additional assays would be needed to confirm this assumption. We, therefore, plan to expand our PDCO analysis with the addition of markers such as p63 and HMWCK. The integrated capacity within our assay platforms would enable such expansion by further multiplexing currently established assays.

Our multi-parameter analysis also demonstrated that ex vivo prostate PDCOs generated from organ-confined tumor tissue maintain AR activity in culture. Expression of AR, PSA and PSMA protein remained detectable and comparable to matched native tissue samples. The AR-pathway activity is a key driver of early PC pathogenesis; therefore, AR function is indispensable in a model that accurately reflects early PC tumorigenesis. However, recent studies of primary prostate spheroid cultures reported a biomarker shift under ex vivo conditions including a lack of AR mRNA expression, sporadic expression of PSA and diminishing PSMA [8]. Linxweiler et al. reported lack of PSA expression in PDCOs but the secreted protein was detectable in all culture medias [4]. Therefore, conventional analytic techniques might not reach the sensitivity to detect these biomarkers accurately. The integrated analytic capabilities of the Stacks microfluidic platform offer a novel approach to simultaneous detection of prostate-specific biomarkers.

A key advantage of this microscale technology is the ability to perform in-chip buffer-exchanges and staining of live cells without material transfers and pre-processing. In addition to providing quantitative information on cellular distribution and heterogeneity, live cell imaging also provides a unique insight into the structure and organization of cell populations within the PDCOs. Through confocal microscopy imaging, we were able to identify the location and distribution of the various cellular populations comprising the PDCO. Furthermore, Stacks allows for direct evaluation of 3D structure and co-expression patterns within PDCOs. We propose that this approach to PDCO analysis has an improved ability to evaluate AR pathway activity and prostate biomarker expression. The capabilities of the integrated platform utilized in this study also allow for Rapid Drug Cultures and comprehensive, in situ assessment of drug-response in small amounts of patient-derived specimen. Response to both Bicalutamide and Docetaxel treatment showed a significant increase in cell death in our model PDCO cultures in accordance with previous findings [4]. In future cohorts, drug sensitivity should be further assessed at various compound concentrations and lengths of treatment. Furthermore, transcriptomic analysis could be integrated as an alternative measurement of treatment response and genomic analysis to explore potential drug resistance mechanisms. The ability to generate multi-parameter read-outs and replicates within a very limited sample size offers a powerful tool for translational studies using patient-derived specimens.

In establishing PC PDCO cultures, sampling accuracy of tumor foci, optimal tumor-content of the starter specimen and timely processing of tissue specimen are key factors [4]. However, identification of tumor tissue by gross examination has limited sensitivity and specificity, frequently resulting in the inclusion of non-malignant cells [25]. Non-malignant epithelial cells have been shown to overgrow in hormone-naïve primary PDCOs, thereby limiting the utility of these cultures [23]. Confirmation of tumor content is therefore important to establish an accurate primary PC model. In our current study, we developed a novel approach to confirm the presence of tumor cells in tissue specimens. We have shown that prostate carcinoma-associated epigenetic biomarkers including *GSTP1*, *RASSF1*﻿, *RARb* and APC are detectable in our cultures to confirm that these primary PDCOs indeed originated from tumor cells. We selected this approach since hypermethylation of *GSTP1*, *RASSF1*, *RARb* and APC are well-established markers of malignant transformation in prostate tissue [27–30, 38] and since this assay can be performed on limited cellular material. However, the addition of orthogonal markers of malignancy would increase the sensitivity and specificity of this assay as well as provide further confirmation of the malignant nature of the PDCOs. We are therefore working to add AMACR and p63 to our flow cytometry panel and are also planning to include global genomic and transcriptomic analyses of our PDCOs to evaluate for tumor-specific molecular alterations such as ERG, AR splice-variants, and NEPC markers.

To our knowledge, this is the first study to establish multiparameter staining of live prostate PDCOs for direct confocal imaging and microscale epigenetic screening of primary prostate specimen. Overall, our data suggest that this prostate PDCO model faithfully recapitulates the complexity of multifocal PC using orthogonal analyses. Furthermore, we propose to leverage the Stacks microfluidic device as a translational analytical platform for rapid and comprehensive interrogation of phenotypic and molecular endpoints with the capacity to incorporate a complex tumor microenvironment. Collection of such data sets of primary tumor specimen are critical to broaden our knowledge of PC pathogenesis and aid biomarker discovery to refine patient stratification and match disease with more efficient therapeutic modalities.

## Supplementary Information

Below is the link to the electronic supplementary material.

Supplementary file1 (PDF 1906 kb)

## Data Availability

The datasets generated during the current study are available from the corresponding author on reasonable request.
