# Supplementary material for: Live cell molecular analysis of primary prostate cancer organoids identifies persistent androgen receptor signaling
Source: Med Oncol. 2021 Sep 28;38(11):135. doi: 10.1007/s12032-021-01582-y (PMC8478748; doi:10.1007/s12032-021-01582-y)
Supplement: Supplementary file 1 — Supplementary file1 (PDF 1906 kb) [file 12032_2021_1582_MOESM1_ESM.pdf]

ONLINE RESOURCE

Medical Oncology

Live cell molecular analysis of primary prostate cancer organoids identifies persistent Androgen Receptor signaling

Erika Heninger[1], David Kosoff[2], Tamara S. Rodems[1], Nan Sethakorn[2], Anupama Singh[1], Harshitha Gungurthi[1], Kristin Carlson[1], Bing Yang[3], Cole Gilsdorf[1], Cheri A. Pasch[1], Dustin A. Deming[2], Leigh Ellis [4], David J. Beebe[5], David F. Jarrard[3] and Joshua M. Lang[1,2].

[1]University of Wisconsin Carbone Cancer Center, Madison, 1111 Highland Ave., [2]Department of Medicine, University of Wisconsin, Madison, 1111 Highland Ave., Madison, WI 53705, [3]Department of Urology, 1111 Highland Ave., Madison, WI 53705, [4]Division of Medical Oncology, Department of Medicine, Samuel Oschin Comprehensive Cancer Institute, Cedars-Sinai Medical Center, 8700 Beverly Blvd, Los Angeles, CA 90048 [5]Department of Pathology and Laboratory Medicine, 1111 Highland Ave., Madison, WI 53705

To whom correspondence should be addressed: Joshua M. Lang, Email: jmlang@medicine.wisc.edu

**Fluorescent Microscopy**		
**marker**	**fluorochrome**	**purpose**
Hoechst	Hoechst 33342	Nuclear stain
EpCAM	Alexa Fluor™ 488	Epithelial marker
PSMA	PE	Prostate Specific Membrane Antigen
CD49f	Alexa Fluor™ 647	Epithelial stratification
CD45	PE	Leukocyte common antigen
CD49a	Alexa Fluor™ 647	Stromal marker
Androgen Receptor	n/a	AR-pathway marker
Donkey Anti-Rabbit IgG	PE	Secondary antibody
PSA	Cy5	Prostate Specific Antigen
Live/Dead	Image-IT™ DEAD Green™	Cell death detection

Table S1 Multi-color immune fluorescent labeling assay reagents for confocal microscopy

**Flow Cytometry Assay I**		
**marker**	**fluorochrome**	**purpose**
Live/Dead fixable	Ghost Dye™ 510	Dead exclusion; reduce autofluorescence
Fc Block™	n/a	Reduce Fc-mediated antibody binding
CD45	redFluor™ 710	Leukocyte common antigen
CD14	redFluor™ 710	Monocyte/macrophage marker
EpCAM	Brilliant Violet™ 650	Epithelial marker
TROP2	PE	Epithelial marker
CD49f	PerCP Cy5.5	Epithelial stratification
Androgen Receptor	n/a	AR-pathway marker
Donkey Anti-Rabbit IgG	Alexa Fluor™ 488	Secondary antibody
PSMA	PE-Cy7	Prostate Specific Membrane Antigen
PSA	Cy5	Prostate Specific Antigen
CD44	Brilliant UltraViolet™ 737	Stemness marker
CD56	Brilliant UltraViolet™ 650	Neuroendocrine marker

Table S2 Flow Cytometry antibodies utilized in Assay I

**Flow Cytometry Assay II**		
**marker**	**fluorochrome**	**purpose**
Live/Dead fixable	Ghost Dye™ 510	Dead exclusion; reduce autofluorescence
Fc Block™	n/a	Reduce Fc-mediated antibody binding
CD45	PE	Leukocyte common antigen
EpCAM	Alexa Fluor™ 488	Epithelial marker
CD49a	PE-Cy7	Stromal marker

Table S3 Flow Cytometry antibodies utilized in Assay II

Gene	Forward Primer (5' - 3')	Internal Probe (5' - 3')	Reverse Primer (5' - 3')
**GSTP1**	TTCGCTGCGCACACTTC	CGGTCCTCTTCCTGCTGTCTGTTT	CTTTCCCTCTTTCCCAGGTC
**RASSF1**	CCTCCAGAAACACGGGTA	TTTGCGGTCGCCGTCGTTGT	CTTCCTTCCCTCCTTCGTC
**APC**	TTATTACTCTCCCTCCCACCTC	TCTTGTGCTAATCCTTCTGCCCTGC	TGGCAGTTGACACGCATAG
**RARB**	GAAGGAGAACTTGGGATCTT	TTTCCAGGCTTGCTCGGCCAATC	AGCCTGTAATTGATCCAAATGA
**LINE1**	CGCAGGCCAGTGTGTGT	CCGTGCGCAAGCCGA	TCCCAGGTGAGGCAATGC

Table S4 Primer and probe sequences used in qRT-PCR and pre-amplification of enriched methylated DNA

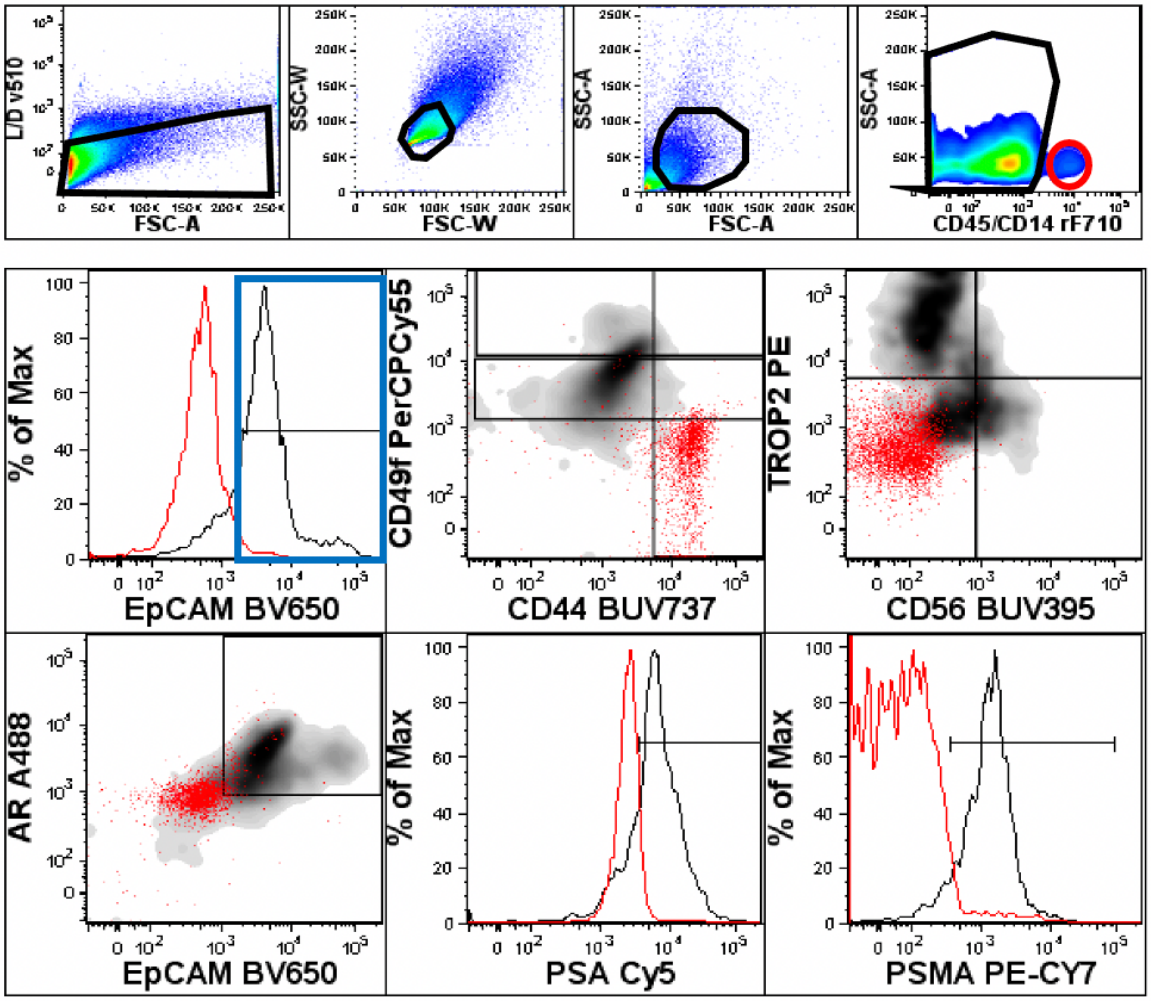

Figure S1 Flow Cytometry Gating Strategy. The live single epithelial cell content was extracted after excluding dead cells (live/dead stain negative; top row first plot), doublets (pulse Width low; top row second plot) and small cells/debris (top row third plot). EpCAM expression was then analyzed after exclusion of CD45$^+$ and CD14$^+$ immune cells (top right, red circle). The CD45$^+$/CD14$^+$ cells served as an Internal Negative Control (red histogram overlay) to establish EpCAM expression in the CD45$^-$/CD14$^-$ fraction (black histogram overlay (second row left). The EPCAM$^+$ cells (blue box on histogram) were then projected for analysis of epithelial and AR-pathway related features as shown in density plots (second row, middle, right) and on histograms (third row middle, right). AR expression is shown on the total CD45$^-$/CD14$^-$ fraction on the density plot on bottom left. The red populations on both density plots and histograms represent the CD45$^+$/CD14$^+$ INC control to confirm gating thresholds

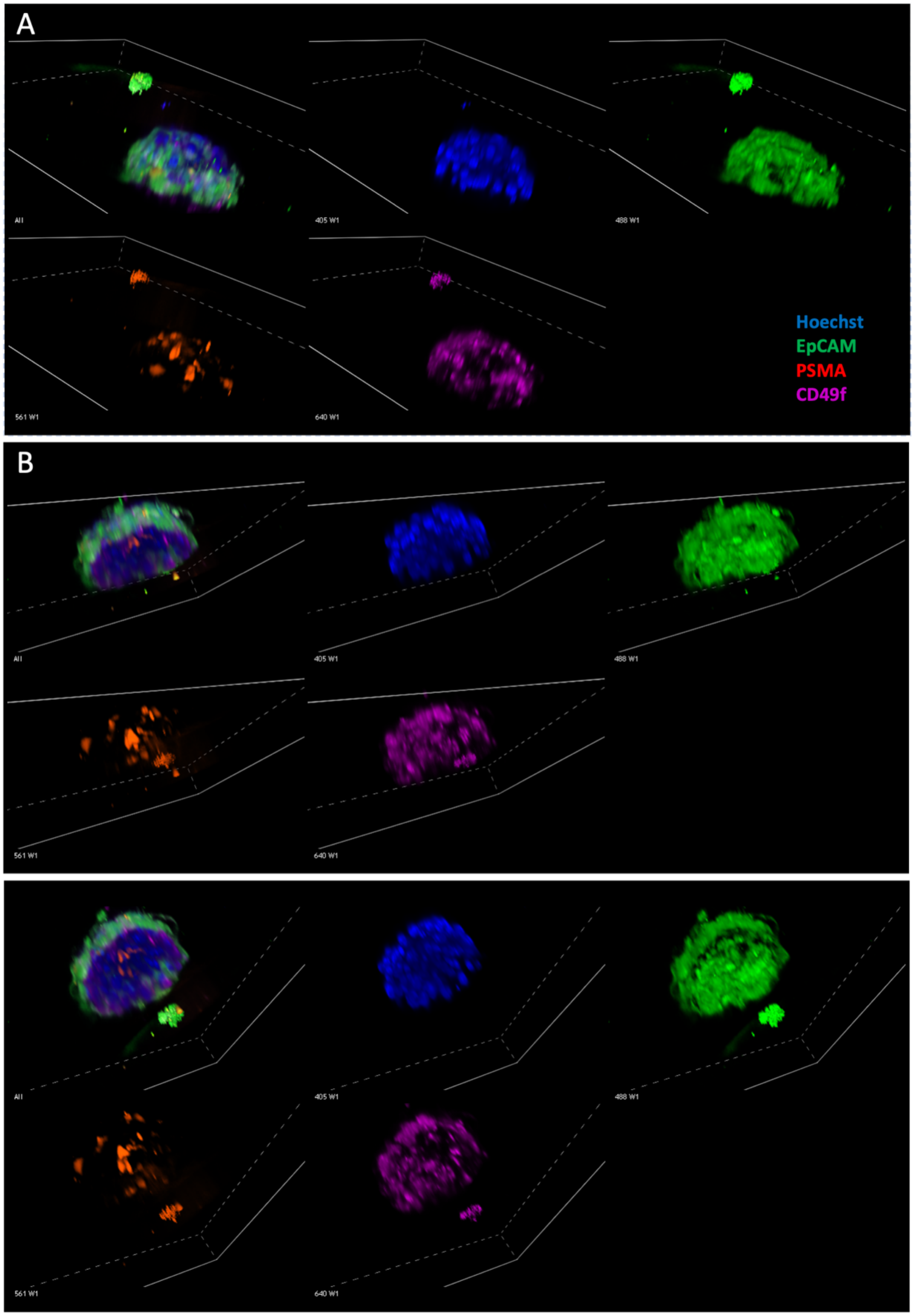

Figure S2. Three-dimensional images of prostate PDCO. Condensed three-dimensional images of PDCO shown in three different angles (a, b,c, respectively). Hoechst (blue), EpCAM (green), PSMA (red) and CD49f (purple).

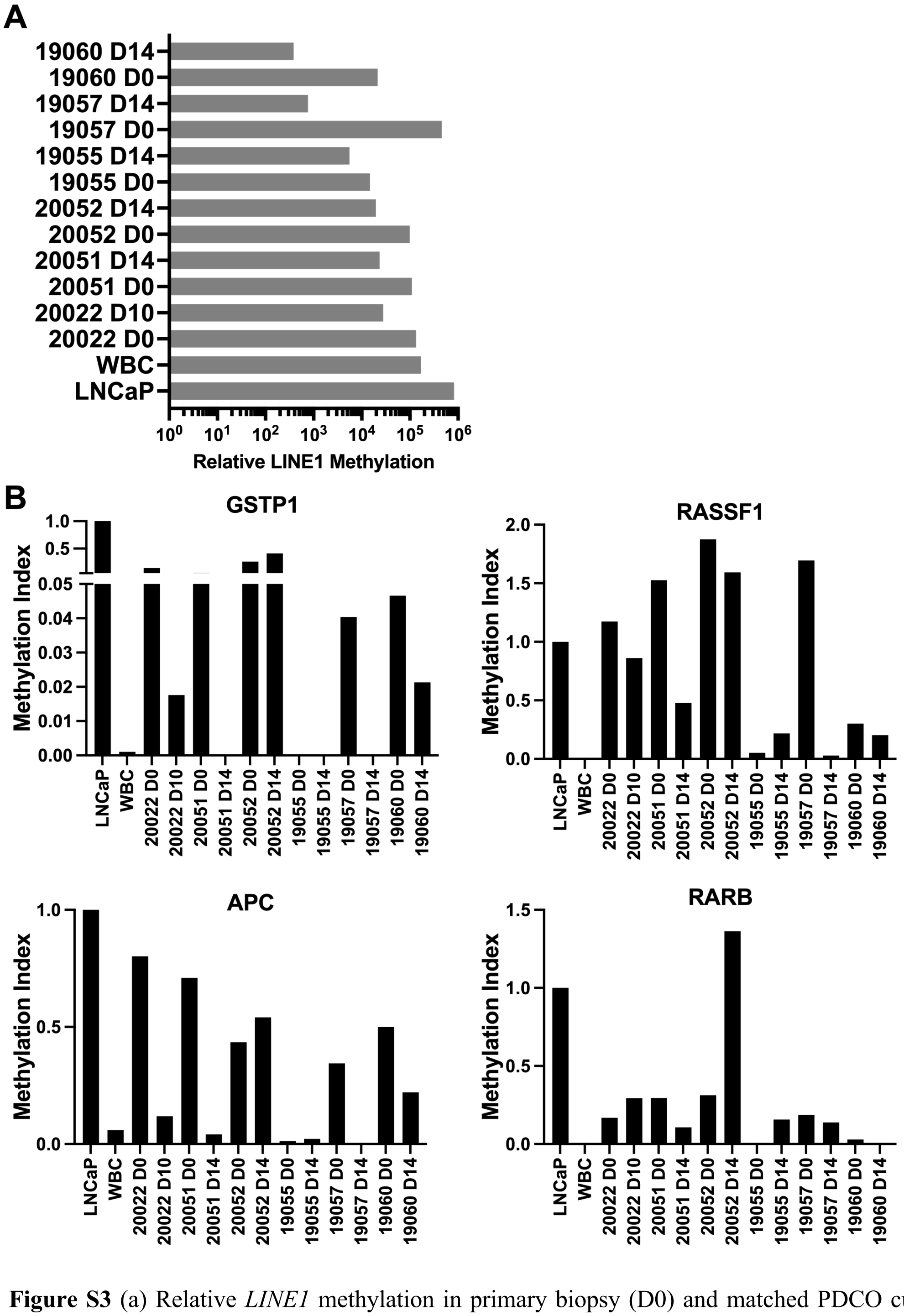

Figure S3 (a) Relative *LINE1* methylation in primary biopsy (D0) and matched PDCO cultures (D10 or D14) after 10-14 days of *ex vivo* culture. LNCaP and WBC *LINE1* methylation is also included. (b) Methylation Index for *GSPT1*, *RASSF1*, *APC*, and *RARB* in primary biopsy (D0) and matched PDCO cultures (D10 or D14) after 10-14 days of culture. Data is the same as Figure 5 and represents individual core punches from radical prostatectomy specimens from the same donor. LNCaP and WBC represent positive and negative controls for promoter methylation at the genes, respectively
